# A model for priority setting of health technology assessment: the experience of AHP-TOPSIS combination approach

**DOI:** 10.1186/s40199-016-0148-7

**Published:** 2016-04-11

**Authors:** Mohammadreza Mobinizadeh, Pouran Raeissi, Amir Ashkan Nasiripour, Alireza Olyaeemanesh, Seyed Jamaleddin Tabibi

**Affiliations:** Department of Health Services Management, Science and Research Branch, Islamic Azad University, Tehran, Iran; Department of Health Services Management, School of Management and Medical Information Science, Iran University of Medical Sciences, Tehran, Iran; National Institute for Health Research, Tehran University of Medical Sciences, Tehran, Iran

**Keywords:** Priority setting, Health Technology Assessment, Iran

## Abstract

**Background:**

In recent times, the use of health technologies in the diagnosis and treatment of diseases experienced considerable and accelerated growth. The goal of the present study was to describe the designated pilot MCDM (Multiple Criteria Decision Making) model for priority setting of health technology assessment in Iran.

**Methods:**

Relevant articles were sought and retrieved from the most appropriate medical databases, including the Cochrane Library, PubMed and Scopus via three separate search strategies, using MESH and free text until March, 2015. Retrieved criteria were questioned from health technology assessment experts in two rounds and the relative weight for valid criteria was finally obtained from paired wise comparison method. After extraction of relative weights based on the aforementioned procedure, TOPSIS (The Technique for Order of Preference by Similarity to Ideal Solution) priority setting model was designed. The stated model was applied for assessing three technologies (adenosine, tissue plasminogen activator and mechanical thrombectomy) which were available for projects call of Iranian health technology assessment department in order to determine applicability of the model for practical purpose.

**Results:**

Nine criteria, including efficiency/effectiveness, safety, population size, vulnerable population size, availability of alternative technologies, cost effectiveness in other countries, budget impact, financial protection, quality of evidence, were extracted by the Iranian health technology assessment experts. The relative weights of these criteria were as follows 0.12, 0.2, 0.06, 0.08, 0.08, 0.13, 0.08, 0.09, and 0.15, respectively. Finally TOPSIS pilot model was designed by three health technologies and nine criteria relative weights. Results showed that, the applicability of the stated model was suitable and as the pilot testing, tissue plasminogen activator was the first priority, adenosine was second and mechanical thrombectomy was third for performing health technology assessment by the Iranian ministry of health and medical education.

**Conclusion:**

According to the results of this study, this model with nine effective criteria and their relative weights and in combination with TOPSIS approach could be used with suitable applicability by health technology assessment department in deputy of curative affairs and food and drug organization for determination of research priorities in health technology assessment.

## Background

Acceptance of health technologies in health systems within the last decades has experienced some intensive changes. The belief in abiding with the acceptance of health technologies within 1960s and 1970s gradually was replaced by an increasing suspicion of society towards the credibility of advanced health technologies. Nowadays, politicians and the public have an ambiguous opinion about the use of a new health technology [1].

Within recent years, the use of health technologies for diagnosis and treatment of diseases experienced considerable and accelerated growth. The proper use of these technologies may considerably help in the diagnosis and treatment of diseases. On the other hand, unlimited and unrestricted entry of these technologies may result in induced demand by service providers [2].

Given, the number of health technologies which required assessment was more than available resources; so all health technology assessment organizations must prioritize their research projects [3]. Various studies have been applied for this purpose worldwide until now, such as Canadian Drug and Health Technologies Organization (CADTH) which utilized the analytical hierarchy process (AHP) for priority setting of health technologies [4]. Another existing model in relation to priority and selection of health technologies was EVIDEM (Evidence and Value: Impact on Decision Making) model in Canada [5]. Nonetheless, there is no consensus on appropriate priority setting between health technology assessment organizations [3]. The main goal of the present study was to describe the designated pilot MCDM model for priority setting of health technology assessment in Iran.

## Methods

In order to achieve the aforementioned goal, relevant articles until March, 2015, based on three separate strategies using MESH and free text were sought for using the Cochrane Library, Pub Med and Scopus. The criteria and sub criteria (in pre assessment and assessment phase, at this stage, the criteria of assessment phase were also included, on the assumption that there may be some of these criteria which can be used in pre assessment phase in Iranian health system context) obtained from systematic review were questioned by experts of health technology assessment context via questionnaire (these expert were selected from official committee of HTA in Iranian ministry of health and medical education and during of this research just 11 ones of them were available for inclusion). At first, to determine the content validity as pilot, experts of health technology assessment were provided with questionnaire in three forms, including (a.) determination of face validity for each item (as two-options: it is clear and demonstrative or it is not clear and demonstrative), (b.) determination of content validity rate (CVR) for each item (as three-options: it is necessary, useful but not necessary or not necessary) and (c.) determination of content validity index (CVI) for each item (as five-options: completely irrelevant, irrelevant, relatively irrelevant, relevant or completely relevant). Sub criteria from which the minimum content validity was obtained was also included in the final questionnaire phase. In this questionnaire, the importance of each of these sub criteria was questioned by experts in these five options context (very slightly important, slightly important, moderately important, important, and very important). For this purpose, a 75 % cut point was used. This implied that for each one of these sub criteria, 75 % of experts who voted important or very important in the process of health technology assessment priority setting were included in the analytic hierarchy process (AHP) phase for extraction of their relative weight through paired wise comparisons. In the analytical hierarchy process, relative weight was obtained by paired comparison matrix. For a consistent paired comparison matrix, weight was calculated through normalization of elements of each column. However, if the matrix was inconsistent, to obtain the weight, four major methods were introduced, including 1 - least squares, 2 - least logarithm squares, 3 - Eigen vector, 4 - approximate methods [6]. The first three methods involve tedious calculations, thus some approximate methods have been proposed which are of lower accuracy (that is acceptable) and involve less and simpler calculations. In this study, geometric mean method was used. The geometric mean of elements of each row was calculated; later, the obtained vector was normalized to obtain the weight vector. Inconsistency rate of each matrix A was calculated based on the following steps:

1. Formation of the paired comparison matrix A;
2. Specification of the weight vector (W);
3. Is the maximum Eigen value of matrix A (*λ*_max_) specified? If yes, go to forth step, otherwise (according to following steps) estimate its value.
  3-1: Obtaining a well-suited estimation of *λ*_max_W by multiplying vector W by matrix A (AW = *λ*_max_W);
  3-2: Upon dividing the obtained values for *λ*_max_W by relative W, estimation of of *λ*_max_ is calculated;
  3-3: The obtained mean *λ*_max_ is calculated;
4. Calculation of inconsistency index (I.I) based on following equation:Formula (1)
  $$ I.I=\frac{\lambda_{\max }-n}{n-1} $$
5. Calculation of inconsistency ratio (I.R.) based on following formula:Formula (2)
  $$ I.R=\frac{I.I}{I.I.R.} $$

In this formula I.I.R. denotes the random matrix inconsistency index. This matrix was calculated for matrices whose numbers were completely randomly selected. In each matrix, the product of dividing the inconsistency index by random matrix inconsistency matrix with the same dimension will be a suitable criterion for judgment about matrix inconsistency which is called inconsistency ratio. If this number is smaller or equal to 0.1, system consistency is acceptable; otherwise the judgments must be revised [6].

After extraction of relative weights based on the above procedure, TOPSIS priority setting model was designed. Essential principles of this method are as follows:

- Appropriateness of each index must be uniform in ascending (descending) order (whatever r_ij_ is higher, appropriateness also higher or vice versa) so that the best existing value of an index denotes its ideal and the worst existing value thereof shows negative ideal for it.
- The interval of an option from ideal (or negative ideal) may be calculated as Euclidean distance (from second exponent) or as the sum total of absolute value of linear distances (known as block gaps) that is dependent on the exchange and replacement rate among indices.

First step: this involves the conversion of existing decision making matrix to a matrix descaled using following formula:

Formula (3):

$$ {n}_{\mathrm{i}\mathrm{j}}=\frac{{\mathrm{r}}_{ij}}{\sqrt{{\displaystyle {\sum}_{\mathrm{i}=1}^{\mathrm{m}}{\mathrm{r}}_{\mathrm{i}\mathrm{j}}^2}}} $$

Second step: this involves creating weighted descaled matrix by assumption of vector W as input to the algorithm.

Formula (4):

$$ \begin{array}{c}\hfill W=\left\{{w}_1,{w}_2,\dots {w}_n\right\}\approx \left(\mathrm{supposed}\ \mathrm{of}\kern0.5em DM\right)\hfill \\ {}\hfill \mathrm{V}={\mathrm{N}}_{\mathrm{D}}.\kern0.5em {\mathrm{W}}_{\mathrm{n}\times \mathrm{n}}=\begin{array}{ccc}\hfill {\mathrm{V}}_{11\dots}\hfill & \hfill {\mathrm{V}}_{1\mathrm{j}\dots}\hfill & \hfill {\mathrm{V}}_{1\mathrm{n}\dots}\hfill \\ {}\hfill \vdots \hfill & \hfill \vdots \hfill & \hfill \vdots \hfill \\ {}\hfill {\mathrm{V}}_{\mathrm{m}1\cdots}\hfill & \hfill {\mathrm{V}}_{\mathrm{m}\mathrm{j}\cdots}\hfill & \hfill {\mathrm{V}}_{\mathrm{m}\mathrm{n}\cdots}\hfill \end{array}\hfill \end{array} $$

Where N_D_ is a matrix having the score of indices therein descaled and comparable and W_n × n_ is a diametrical matrix having only its main elements as nonzero.

Third step: This involves determination of ideal solution and negative ideal solution

For ideal option A^+^ and negative ideal A^-^, the following definition is provided:

Formula (5):

$$ \begin{array}{c}\hfill {\mathrm{A}}^{+}=\left\{\left(\begin{array}{c}\hfill max\hfill \\ {}\hfill i\hfill \end{array}{V}_{ij}\left|j\in J\right.\right),\left(\begin{array}{c}\hfill min\hfill \\ {}\hfill i\hfill \end{array}{V}_{ij}\left|j\in J\right.\right)\left|i=1,2,\dots m\right.\right\}=\left\{{V}_1^{+},{V}_2^{+},\dots, {V}_j^{+},\dots, {V}_n^{+}\right\}\hfill \\ {}\hfill {\mathrm{A}}^{\hbox{-} }=\left\{\left(\begin{array}{c}\hfill min\hfill \\ {}\hfill i\hfill \end{array}{V}_{ij}\left|j\in J\right.\right),\left(\begin{array}{c}\hfill max\hfill \\ {}\hfill i\hfill \end{array}{V}_{ij}\left|j\in J\right.\right)\left|i=1,2,\dots m\right.\right\}=\left\{{V}_1^{-},{V}_2^{-},\dots, {V}_j^{-},\dots, {V}_n^{-}\right\}\hfill \\ {}\hfill J=\left\{j=1,2,\dots, n\left|\mathrm{j}\mathrm{s}\right.\kern0.5em \mathrm{referred}\kern0.5em \mathrm{t}\mathrm{o}\kern0.5em \mathrm{t}\mathrm{he}\kern0.5em \mathrm{profit}\right\}\hfill \\ {}\hfill J=\left\{j=1,2,\dots, n\left|\mathrm{j}\mathrm{s}\right.\kern0.5em \mathrm{referred}\ \mathrm{t}\mathrm{o}\kern0.5em \mathrm{t}\mathrm{he}\kern0.5em \mathrm{cost}\right\}\hfill \end{array} $$

Forth step: This involves calculation of separation size (distance).

The distance between option i and ideals based on Euclidean method is as follows:

Formula (6)

$$ \begin{array}{c}\hfill {\mathrm{d}}_{\mathrm{i}+}={\left\{{\displaystyle \sum_{\mathrm{j}=1}^{\mathrm{n}}{\left({\mathrm{V}}_{\mathrm{i}\mathrm{j}}\hbox{-} {\mathrm{V}}_{\mathrm{j}}^{+}\right)}^2}\right\}}^{0.5},\mathrm{i}=1,2,\dots, \mathrm{m}\hfill \\ {}\hfill {\mathrm{d}}_{\mathrm{i}\hbox{-} }={\left\{{\displaystyle \sum_{\mathrm{j}=1}^{\mathrm{n}}{\left({\mathrm{V}}_{\mathrm{i}\mathrm{j}}\hbox{-} {\mathrm{V}}_{\mathrm{j}}^{\hbox{-}}\right)}^2}\right\}}^{0.5},\mathrm{i}=1,2,\dots, \mathrm{m}\hfill \end{array} $$

Fifth step: This involves calculation of relative closeness of A_i_ to the ideal solution. This relative closeness is defined as follows:

Formula (7):

$$ c{l}_{i+}=\frac{d_{i-}}{\left({d}_{i+}+{d}_{i-}\right)};0\le c{l}_{i+}\le 1;i=1,2,\dots, m $$

As observed, if

$$ {A}_i={A}^{\mp } $$

Then:

$$ {d}_{i+}=0 $$

And will have:

$$ c{l}_{i+}=1 $$

And in case:

$$ {A}_i={A}^{-} $$

Then:

$$ {d}_{i-}=0 $$

And

$$ c{l}_{i+}=0 $$

Therefore, whatever A_i_ is closer to the ideal solution A^+^, value of cl_i_ will be closer to the unit.

Sixth step: This involves ranking of the options. The existing options of supposed problem can be ranked based on the descending order *cl*_*i* +_ [7, 8].

Ultimately, the first designed model was examined by pilot test using the last health technology assessment projects call of The Iranian Department of Health Technology Assessment, including 3 technologies (Adenosine, Tissue Plasminogen Activator (tPA), Mechanical Thrombectomy) and the results were provided to the experts for judgment.

## Results

### Systematic review

After screening of retrieved papers via PRISMA framework, from 7012 papers, 40 were left included in the final phase. Criteria for selection of health technologies (in pre-assessment and also in assessment phase) were categorized into the six main themes such as 1 - health outcomes, 2 - disease and target population, 3 - technology alternatives, 4 -economic aspects, 5 - evidence and 6 - other factors. Maximum frequency in health outcomes related to health effects/benefits (8 studies), maximum frequency in disease and target population was related to disease severity (12 studies), maximum frequency in alternatives was related to number of alternatives (2 studies), maximum frequency in economic aspects was related to cost-effectiveness (15 studies), maximum frequency in evidence was related to quality of evidence (4 studies) and in other factors, max frequency was related to issues concerning the health system (11 studies) (Table 1).

**Table 1:** Classification of main criteria and sub criteria based on frequency in reviewed Studies (in pre-Assessment and also in Assessment phase)

Main Criteria	Health Outcomes		Disease and Target Population		Alternatives		Economic Aspects		Evidence		Other Factors	
	Sub criteria	Frequency	Sub criteria	Frequency	Sub criteria	Frequency	Sub criteria	Frequency	Sub criteria	Frequency	Sub criteria	Frequency
1	Health effects/benefits	8	Disease severity	12	Number of alternatives	2	Cost-effectiveness	15	Quality of evidence	4	Issues related to health system	11
2	Clinical effects/benefits	7	Disease burden	8	Availability of alternative	1	Costs	11	Number of evidence	3	Sporadic sub criteria	11
3	Efficacy/effectiveness	4	Target population age	7	Limitations of comparative interventions	1	Budget impact	10	Evidence relevance and validity	1	Political, social and moral issues	10
4	Individual health benefits	3	Population size	3	Lack of alternatives	1	Economic impact	6	Power of evidence	1	Benefits of beneficiaries	6
5	Safety	3	Number of potential beneficiaries	3	-	-	Poverty Reduction	4	Completeness and consistency of reporting evidence	1	Issues related to patients	5
6	Quality of life	1	Characteristics of target social groups for intervention	2	-	-	Value for money	2	Adherence to requirements of decision making body	1	Issues related to decision-making conditions	5
7	Potential changes in health consequences	1	Number of patients	2	-	-	Financial opportunity/consequences	2	-	-	Fairness and equity	4
8	The effect of assessment on reduction of uncertainty	1	Effect of technology on reduction of disease prevalence and incidence	1	-	-	Economic productivity	2	-	-	-	-
9	Marginal benefits	1	Disease impacts	1	-	-	Financial protection	1	-	-	-	-
10	Ability to reduce own health risk	1	Effect on targeted groups	1	-	-	Subsidized Payment	1	-	-		
11	Potential to extend life	1	Size of vulnerable population	1	-	-	Society interest and demand	1	-	-	-	-
12	Potential to detect a condition which, if treated early, averts costs in the future	1	-	-	-	-	Technology price and sale volume	1	-	-	-	-

### Phase 1: Content validity verification

Sub criteria obtained from the systematic review phase that were classified into six dimensions were designed as three questionnaires, including 54 sub criteria and one question for extraction of extra criteria. To determine the content validity of 11 health experts, technology assessment were questioned in three ways: (a.) determination of face validity for each item (as two-option: it is clear and demonstrative or it is not clear and demonstrative), (b.) determination of content validity ratio (CVR) for each item (as three-option: it is necessary, useful but not necessary or not necessary) and (c.) determination of content validity index (CVI) for each item (as five-option: completely irrelevant, irrelevant, relatively irrelevant, relevant or completely relevant). Firstly, content validity index (CVI) was calculated for each item such that out of 55 items, 21 items were obtained following a minimum of 0.79 which included health benefits, clinical impacts, efficacy/effectiveness, potential to increase the life, safety, population size, the number of potential beneficiaries, the impact on target groups, the size of vulnerable populations, the availability of alternative technologies, cost-effectiveness, costs, budget impact, financial protection, the quality of evidence, number of evidence, the issues related to the health system, the issues related to the availability of expertise, equity in better access to health care and the question of additional criteria. Shortly following this, content validity ratio was calculated for each item such that, out of 54 sub criteria, all questioned experts had consensus on the necessity of only 9 sub criteria (health benefits, clinical benefits, efficacy/effectiveness, safety, cost-effectiveness, costs, technology price, quality of the evidence and issues related to rate availability of expertise) in this framework and obtained a score higher than score of Lawshe table for 11 experts. Whereas, Lawshe clarified that each item with more than half the total population of people had consensus on its necessity, and had a level of content validity (to the same rate of people that voted to its necessity) [9]. Thus, in addition to 9 sub criteria that had obtained the required score of CVR, a sub criteria greater than 50 % of people (including 50 % itself and higher) voted to its necessity and a CVI greater than 0.79 was obtained, kept and included into the final questionnaire (for assessment of their importance). These sub criteria were as follows: clinical impacts, efficiency/effectiveness, individual health benefits, safety, population size, number of potential beneficiaries, effect on targeted group, vulnerable population size, availability of alternative technologies, quality of evidence, number of evidence, budget impact, cost effectiveness, financial protection, issues related to health system and availability rate of experience and specialty and additional question for other criteria. Based on face validity, according to the opinion of experts, 4 sub criteria including individual health benefits, clinical impacts, efficiency/effectiveness, and effect on targeted group due to their overlap were all combined in the efficiency/effectiveness sub criteria. In addition, in the same manner, sub criteria of number of potential beneficiaries was combined with the population size, costs combined with cost effectiveness while number of evidence was renamed as the adequacy of the evidence. Thus, in the final questionnaire, 13 sub criteria were included based on 6 dimensions of conceptual model of study (Table 2).

**Table 2:** Sub criteria obtained after first phase of question from experts

No.	Criteria	Sub criteria
Dimension 1	Health Outcomes	Efficiency/Effectiveness, Safety
Dimension 2	Disease and target population	Population Size, Vulnerable Population Size
Dimension 3	Alternatives	Availability of Alternative Technologies
Dimension 4	Economic Aspects	Budget Impact, Cost Effectiveness of Technology in Other Countries, Financial Protection
Dimension 5	Evidence	Quality of Evidence, Adequacy Number of Evidence
Dimension 6	Other criteria	Issues Related to Health System, Issues Related to Availability of Experience and Specialty about Technology and Issue Related to the Equity

### Phase 2: Determining importance of each sub criteria

Thirteen obtained sub criteria in previous phase were included into the final questionnaire phase based on the same process, 9 sub criteria, including Efficiency/effectiveness, safety, Population size, vulnerable population size, availability of alternative technologies, budget Impact, cost effectiveness of technology in other countries, financial protection, quality of evidence that obtained a greater than 75 % importance as viewpoint of questioned experts, were included in the paired wise comparisons questionnaire as the final 9 effective criteria in the priority setting of health technology assessment and this finalized the extraction of relative weight (Table 3). To assess the reliability of the final questionnaire, data obtained from each sub criterion were entered into SPSS software as Likert spectrum and the Cronbach’s alpha was calculated with its value at 0.697. This demonstrated an acceptable reliability of the final questionnaire. Moreover, no consensus was made between questioned experts in relation to the additional criteria individually mentioned by the experts. These criteria include Ethical issues, burden of disease, information value, the elasticity of demand for technology, procedures and methods of diagnosis and treatment, the external dependence of the use of technology, the development and application of technology in the world and the indication of technology.

**Table 3:** Final sub criteria obtained after two phases questioning from experts

No.	Criteria	Sub criteria
Dimension 1	Health Outcomes	Efficiency/Effectiveness, Safety
Dimension 2	Disease and target population	Population Size, Vulnerable Population Size
Dimension 3	Alternatives	Availability of Alternative Technologies
Dimension 4	Economic Aspects	Budget Impact, Cost Effectiveness of Technology in Other Countries, Financial Protection
Dimension 5	Evidence	Quality of evidence
Dimension 6	Other criteria	-

### Phase 3: Identification of relative weights of final criteria

Nine final effective criteria selected based on the opinion of experts were designed during the first and second phases as paired comparisons questionnaire and in another case they were presented to nine questioned experts in the first and second phases (at this phase, two experts of the previous phases were not available) in order to extract the relative weight of each one of the 9 final criteria. After filling the 9 questionnaires aforementioned, the information of these questionnaires was entered into an excel sheet as 9 matrixes (9*9) and to extract the final weight and equalizing the data of these 9 matrixes, a final matrix was formed such that in each one of its entries was included the geometric mean of its corresponding entries in 9 matrixes (9*9). The relative weigh of each criterion was extracted from the final formed matrix through normalization of geometric mean of each line thereof. Later, the obtained inconsistency ratio for the system was calculated, which was less than the range of 0.1 (IR = 0.03) and demonstrated consistency between extracted weights (Table 4).

**Table 4:** Relative weight obtained from normalization of geometric mean of each row of final matrix

	Efficiency/effectiveness	Safety	Population size	Vulnerable population	Availability rate of alternative technologies	Cost effectiveness in other countries	Budget impact	Financial protection	Quality of evidence
Relative Weight	0.12	0.20	0.06	0.08	0.08	0.13	0.08	0.09	0.15

### Phase 4: Piloting the priority setting model

According to the inquiry from health technology assessment department of ministry of health, and medical education, 3 health technologies that were requested for health technology assessment, were selected and TOPSIS priority setting pilot model was implemented on them. These technologies included the following:

- Adenosine
- Tissue Plasminogen Activator (tPA)
- Mechanical Thrombectomy

Furthermore, to fill the decision matrix for execution of model pilot, values 1 to 5 were designed for each one of 9 final criteria, and data related thereto were extracted from available evidence for the three pilot technologies: (Tables 5, 6, 7, 8, 9, 10, 11, 12 and 13).

**Table 5:** Coding of efficiency/effectiveness criteria [10]

Quantitative code	Status	For Technologies Related to Treatment and Rehabilitation: Clinical Benefit and Improvement of Quality of Life
5	Perfect treatment	
4	Lifetime increasing and major improvement of quality of life	
3	Lifetime increasing and low improvement of quality of life	
2	Major improvement of quality of life	
1	Low improvement of quality of life	
Quantitative code	Status	For Technologies Related to Screening and Diagnosis: Accuracy of Technology and Treatment Status of Disease under Screening and Diagnosis
5	Technology accuracy was more than 80 % and disease under screening and diagnosis may be treated.	
4	Technology accuracy was within 60–80 % and disease under screening and diagnosis may be treated.	
3	Technology accuracy was over 80 % but the disease under screening and diagnosis may not be treated.	
2	Technology accuracy was within 60–80 % and disease under screening and diagnosis may not be treated or accuracy was 60 % and disease under screening and diagnosis may be treated.	
1	Technology accuracy was less than 60 % and disease under screening and diagnosis may be treated.	
Quantitative code	Status	For Technologies Related to Prevention: Effectiveness of Technology for Prevention of Disease
5	Over 90 %	
4	81–90 %	
3	71–80 %	
2	61–70 %	
1	Less or equal to 60 %

**Table 6:** Coding of Safety Criteria (as qualitative estimation or using quantitative indicators available in the safety context): Improvement rate in reduction of risk or incidence of side effects arising out of utilization of technology in the target population

Quantitative code	Status
5	Completely safe (lack of side effects) (Incidence of side effects less than 1 %)
4	Relatively safe (mild and slight side effects) (Incidence of side effects between 1 to 9.9 %)
3	Proper safety (averagely side effects) (Incidence of side effects between 10 to 39 %)
2	Poor safety (much side effects) (Incidence of side effects 40 to 50 %)
1	Unsafe (major side effects) (Incidence of side effects more than 50 %)

**Table 7:** Coding of population size criteria: number of people under effect of disease or health problem that will be treated or prevented by technology [10]

Quantitative code	Status
5	More than 500000
4	100001 to 500000
3	50001 to 100000
2	10001 to 50000
1	Less or equal to 10000

**Table 8:** Coding of vulnerable population criteria: share of children below 5 years old or women in fertility ages or elders over 65 years old or patients suffering from special diseases (including MS, dialysis, hemophilia and thalassemia) or patients suffering from neuropsychological diseases in the target population

Quantitative code	Status
5	More than 50 %
4	26–50 %
3	11–25 %
2	Less or equal to 10 %
1	Unspecified vulnerable population size

**Table 9:** Coding of Availability rate of Alternative technologies (qualitative estimation): Availability of other alternative methods for technology in target population

Quantitative code	Status
5	Partial availability of alternative technologies with the same safety and effectiveness for target population
4	Relatively appropriate availability of alternative technologies with the same safety and effectiveness for target population
3	Appropriate availability of alternative technologies with the same safety and effectiveness for target population
2	High availability of alternative technologies with the same safety and effectiveness for target population
1	Very high availability of alternative technologies with the same safety and effectiveness for target population

**Table 10:** Coding of quality of evidence: quality of evidences of health technology assessment, systematic review and clinical trials on technology

Quantitative code	Status
5	Systematic reviews with high quality of randomized controlled trials or heath technology assessment with high quality therein systematic review has been performed (based on checklist of CASP and INAHTA)
4	Systematic reviews with medium or low quality of randomized controlled trials or heath technology assessment with medium or low quality therein systematic review has been performed (based on checklist of CASP and INAHTA)
3	Randomized controlled trials or clinical controlled trials with high quality or systematic review with high quality of other studies (based on CASP checklist)
2	Randomized controlled trials or clinical controlled trials with medium or low quality or systematic review with medium or low quality of other studies or cohort studies with any kind of quality (based on CASP checklist)
1	Other studies (cohort, case series and quasi-random studies etc.) with any quality

**Table 11:** Coding of cost effectiveness: status of cost effectiveness of technology in other countries

Quantitative code	Status
5	very cost effective (cost per QALY lower than 1 time more than per capital GDP)
4	Reasonable cost effective (cost for QALY within 1–2 times more than per capital GDP)
3	Slightly cost effective (cost for QALY within 2–3 times more than per capital GDP)
2	Not-cost effective (cost for QALY more than 3 times more than per capital GDP)
1	Unspecified cost effectiveness status

**Table 12:** Coding of budget impact (as qualitative estimation): net impact of covering the technology on the budget of health system

Quantitative code	Status
5	Significant savings
4	Moderate savings
3	Very slight change in the amount of savings
2	No significant changes in the costs or additional charges
1	Unspecified budget impact status

**Table 13:** Coding of financial protection: the probability that the technology covered by the basic health insurance package and thus prevent the occurrence of catastrophic health expenditures

Quantitative code	Status
5	100 %
4	76 to 99 %
3	50 to 75 %
2	Less than 50 %
1	Unspecified financial protection status

#### The data obtained from evidence about three above-mentioned technologies

##### Adenosine for the treatment of supraventricular tachycardia [11]

Status of efficiency/effectiveness criteria: This medication with a dose of 12 mg quickly and effectively terminated proximal episodes of the supraventricular tachycardia (relative code = 5) [12]. Status of safety criteria: The use of this medication resulted in a slightly bad side effect such as confusion, nausea, redness of face etc. (relative code = 4) [13]. Status of population size criteria: Supraventricular tachycardia prevalence was 2.25 cases in a general 1000-persons population. Therefore, the size of target population of this medication in Iran, considering its 80 million population was about 180 thousand peoples (relative code = 4) [11]. Status of vulnerable population size criteria: The probability of supraventriclar tachycardia in women was 2 times more than men, and majorly in pregnancy and fertility ages (within 15–30 years) (relative code = 4) [11]. Status of alternative technologies availability criteria: Verapamile is the alternative medication that has equal efficiency and relatively equal safety and mostly available in the health centers (relative code = 2) [11, 13]. Status of quality of evidence criteria: Concerning this medication, there is a systematic review study of randomized controlled studies of meta-analysis type with a high quality (relative code = 5) [13]. Status of cost effectiveness criteria: The data about this criterion was unspecified (relative code = 1). Status of budget impact criterion: The data about this criterion was unspecified (relative code = 1). Status of financial protection criterion: The data about this criterion was unspecified (relative code = 1).

##### Tissue plasminogen activator (tPA) for the treatment of stroke [14]

Status of efficiency/effectiveness criteria: The use of this medication resulted in a 0.07 increase of lifetime together with 0.24 increase of QALY (relative code = 3) [15]. Status of safety status: In comparison to placebo, the use of this technology had higher risk of intracranial hemorrhage (relative code = 3) [16]. Status of population size criteria: prevalence of stroke in west countries was about 12000 per million peoples [17]. The prevalence and incidence of stroke in Iran was significantly higher than western countries [18]. Thus, in Iranian population, assuming 80 million people, the population size under impact of this disease will be higher than one million people (relative code = 5). Status of vulnerable population size criteria: The incident of stroke was mostly prevalent among Iranian women (within 51–53 %) and majorly occurred within seventh decade of life (relative code = 4) [19]. Status of alternative technologies availability criteria: This technology was the first and the only effective therapeutic method for patients with stroke 3 times after its first occurrence (relative code = 5) [16]. Status of quality of evidence criteria: In relation to this technology, there was a high quality randomized multicenter controlled trial (elative code = 3) [16]. Status of cost effectiveness criteria: The incremental cost effectiveness ratio for all patients consuming this medication was about 6255 Dollars per QALY. This value was obtained for USA. Considering her per capital GDP in 2014 as equal to 53000$. This value is rather highly cost effective (relative code = 5) [15]. Status of budget impact criteria: The data in relation to this criterion was unspecified (relative code = 1). Status of financial protection criteria: The data in relation to this criterion was unspecified (relative code = 1).

##### Mechanical thrombectomy for the treatment of stroke [20]

Status of efficiency/effectiveness criteria: This technology resulted in a 0.68 increase of QALY (relative code = 1) [21]. Status of safety criteria: This technology was safe which could demonstrate higher rate of symptomatic bleeding than medicinal methods (relative code = 3) [20]. Status of population size criteria: Prevalence of stroke in west countries was about 12000 per million peoples [17]. The Prevalence and incidence of stroke in Iran was significantly higher than western countries [18]. Thus, in Iranian population, assuming 80 million people, the population size under impact of this disease will be higher than one million people (relative code = 5). Status of vulnerable population size criteria: The incident of stroke was mostly prevalent among Iranian women (within 51–53 %) and majorly occurred within seventh decade of life (relative code = 4) [19]. Status of alternative technologies availability criteria: This method was the only therapeutic method for patients with incident of stroke who were not treated by clot solving medications, since the use of this method resulted in failure with respect to them (during first 3–4 h after this occurrence) (relative code = 5) [20]. Status of quality of evidence criteria: In relation to this technology, there was health technology report from randomized r controlled trial with high quality (elative code = 5) [20]. Status of cost effectiveness criteria: The incremental cost effectiveness ratio for all patients taking this medication was about 16000 Dollars per QALY. This value was obtained for USA and considering per capital GDP of this country in 2014, which equaled 53000$, this value is rather highly cost effective (relative code = 5) [21]. Status of budget impact criteria: The data in relation to this criteria was unspecified (relative code = 1). Status of financial protection criterion: The data in relation to this criterion was unspecified (relative code = 1).

#### Final TOPSIS pilot model

Finally TOPSIS pilot model was designed by 3 health technologies and relative weights. The results showed that tissue plasminogen activator treatment was the first priority, adenosine was second and mechanical thrombectomy was third in performing health technology assessment by Iranian ministry of health and medical education (Table 14). Finally, the pilot model was presented to an internal panel which was made up of 6 experts of health technology assessment and was approved.

**Table 14:** Final TOPSIS pilot model

Decision Matrix									
Criteria	Efficiency/effectiveness	Safety	Population size	Vulnerable population	Availability rate of alternative technologies	Cost effectiveness in other countries	Budget impact	Financial protection	Quality of evidence
Technology									
Adenosine	5	4	4	4	2	1	1	1	5
Tissue Plasminogen Activator	3	3	5	4	5	5	1	1	3
Mechanical Thrombectomy	1	3	5	4	5	5	1	1	5
Weight Matrix									
	Efficiency/effectiveness	Safety	Population size	Vulnerable population	Availability rate of alternative technologies	Cost effectiveness in other countries	Budget impact	Financial protection	Quality of evidence
Relative Weight	0.12	0.20	0.06	0.08	0.08	0.13	0.08	0.09	0.15
Norm Matrix									
Criteria	Efficiency/effectiveness	Safety	Population size	Vulnerable population	Availability rate of alternative technologies	Cost effectiveness in other countries	Budget impact	Financial protection	Quality of evidence
Technology									
Adenosine	0.85	0.69	0.49	0.58	0.27	0.14	0.58	0.58	0.65
Tissue Plasminogen Activator	0.51	0.51	0.62	0.58	0.68	0.70	0.58	0.58	0.39
Mechanical Thrombectomy	0.17	0.51	0.62	0.58	0.68	0.70	0.58	0.58	0.65
V Matrix									
	Efficiency/effectiveness	Safety	Population size	Vulnerable population	Availability rate of alternative technologies	Cost effectiveness in other countries	Budget impact	Financial protection	Quality of evidence
Adenosine	0.11	0.14	0.03	0.05	0.02	0.02	0.05	0.05	0.10
Tissue Plasminogen Activator	0.06	0.10	0.04	0.05	0.05	0.09	0.05	0.05	0.06
Mechanical Thrombectomy	0.02	0.10	0.04	0.05	0.05	0.09	0.05	0.05	0.10
Positive Ideal									
	Efficiency/effectiveness	Safety	Population size	Vulnerable population	Availability rate of alternative technologies	Cost effectiveness in other countries	Budget impact	Financial protection	Quality of evidence
A+	0.11	0.14	0.04	0.05	0.05	0.09	0.05	0.05	0.10
Negative Ideal									
	Efficiency/effectiveness	Safety	Population size	Vulnerable population	Availability rate of alternative technologies	Cost effectiveness in other countries	Budget impact	Financial protection	Quality of evidence
A-	0.02	0.10	0.03	0.05	0.02	0.02	0.05	0.05	0.06
Final Priority Setting									
d1+	0.08	d1-	0.10	closeness1	0.56	2			
d2+	0.07	d2-	0.09	closeness2	0.57	1			
d3+	0.09	d3-	0.09	closeness3	0.49	3			

## Discussion

Generally, this study which was applied based on the combined model of analytic hierarchy process and TOPSIS, was presented for the first time in the world, in the health technology assessment priority setting context. The differences and similarities between this model and main model of Canadian Agency for Drugs and Technologies in Health are explained in terms of finalized criteria in this model. The criteria used in this model which extracted using a systematic review which included a number of alternatives and access such as, budget impact, clinical impact, controversial nature of technology, disease burden, economic impact, moral and legal issues, quality and level of evidences, expected level of interests, time period of assessment and its effect on health and clinical medicine policies, variety in direction for use and application of technology. The criteria used in the model of this study included efficiency/effectiveness, safety, population size, vulnerable population size, availability of alternative technologies, cost effectiveness in other countries, budget impact, financial protection, and quality of evidences. Efficiency/effectiveness, availability of alternative technologies, quality of evidences and budget impact as common items and controversial nature of technology, disease burden, economic impact, moral and legal issues, expected level of interests, time period of assessment and its effect on health and clinical medicine policies, variety in direction for use and application of technology in Canadian model and safety, population size, vulnerable population size, cost effectiveness in other countries and financial protection in the model of this study were assumed as differences. In relation to multi criteria decision making model used in this study, it is noteworthy that in Canadian model, overall relative weight extraction, as well as, final priority setting were performed based on analytic hierarchy process, whilst in the model of this study, only relative weight was extracted for determination of exact weight using final purpose which implied that, health technology assessment priority setting in Iran, through paired comparisons and no judgment of experts, were applied on the final model. This subject was designed in order to adhere to evidence based medicine (EBM) and its evidence pyramid using TOPSIS model and ascertain the use of codes extracted from evidence related to the technology, which can prevent the fully and unlimited interventions of medical beneficiary groups for selection of health technologies that are assumed as the stewardship scope. In fact, out of 11 criteria acquired from systematic review phase of Canadian model and mentioned earlier, 6 criteria including disease burden, clinical impact, alternative technologies, budget impact, economic impact and available evidence were selected as the main criteria by health technology assessment experts of that country. In relation to weight extraction phase, the following values were obtained: disease burden (0.216), clinical impact (0.258), alternative technologies (0.081), budget impact (0.143), economic impact (0.167), and available evidence (0.135) [4]. Furthermore, these values were comparable with the values obtained from the present, study including efficiency/effectiveness (0.12), safety (0.2), population size (0.06), vulnerable population size (0.08), availability of alternative technologies (0.08), cost effectiveness in other countries (0.13), budget impact (0.08), financial protection (0.09), and quality of evidence (0.15). The model designed in this study may also be compared to the health technology assessment priority setting model in Latvia. In this model, research of literature review as well as qualitative study was used in the model design, followed by a test for health technology assessment priority setting. To test the model and method, 3 round Delphi model was implemented using electronic questionnaire. Accordingly, Delphi technique was invented as a method for making consensus in the health technology assessment priority setting for Latvia. Criteria affecting this model included health benefit, evidence, assessment scheduling, expected level of policy makers’ benefits, social, legal and moral concepts [22]. Two criteria of health benefit and evidence were common criteria in the model designed in the present study. The designed model of this study may be compared to the health technology assessment priority setting model in Netherlands. In this model, various methods were used for classification, scoring and weighting the policy making criteria, including actual disease burden, potential health benefit, number of patients, direct intervention costs for each patient, financial consequences of intervention implementation over time and its impact on the health system policies [23]. Three criteria, including health benefit, number of patients and financial consequences were assumed as the three common criteria in the model designed for this study. Overall, the model designed in the present study in comparison to previous models in this context had innovation in the light of effective criteria because of its additional safety, vulnerable population size, cost effectiveness of technology in other countries and financial protection to the previous models. In addition, as mentioned before, technically this is the first time that a combined model of analytic hierarchy process and TOPSIS has been used in this relation.

## Conclusion

According to the results of this study, this model with nine effective criteria and their relative weights and in combination with TOPSIS approach could be used with suitable applicability by health technology assessment department in deputy of curative affairs and food and drug organization for determination of research priorities in health technology assessment. Considering the increasing growth of health technologies, ministry of health and medical education, using this model, can allocate its research budgets to health technologies assessment which are of priority and importance to the health system and society. It may promote the final impact of health technology assessment reports on the macro health policies and optimize the allocation of financial resources of health sector.

### Limitations

This model is not applicable for prioritization of orphan drugs.
